# DLG5 suppresses breast cancer stem cell‐like characteristics to restore tamoxifen sensitivity by inhibiting TAZ expression

**DOI:** 10.1111/jcmm.13954

**Published:** 2018-11-18

**Authors:** Jie Liu, Juan Li, Pingping Li, Yina Jiang, He Chen, Ruiqi Wang, Fang Cao, Peijun Liu

**Affiliations:** ^1^ Center for Translational Medicine The First Affiliated Hospital of Xi'an Jiaotong University Xi'an Shaanxi China; ^2^ Key Laboratory for Tumor Precision Medicine of Shaanxi Province The First Affiliated Hospital of Xi'an Jiaotong University Xi'an Shaanxi China; ^3^ Department of Pathology The First Affiliated Hospital of Xi'an Jiaotong University Xi'an Shaanxi China

**Keywords:** DLG5, stemness, tamoxifen resistance, TAZ

## Abstract

Tamoxifen (TAM) is a primary drug for treatment of estrogen receptor positive breast cancer. However, TAM resistance remains a serious threat to breast cancer patients and may be attributed to increased stemness of breast cancer. Here, we show that discs large homolog 5 (DLG5) expression is down‐regulated in TAM‐resistant breast cancer and cells. DLG5 silencing decreased the sensitivity to TAM and increased the frequency and stemness of CD44^+^/CD24^−^ breast cancer stem cells (BCSCs) and TAZ, a transducer of the Hippo pathway, expression in MCF7 cells while DLG5 overexpression had opposite effects. TAZ silencing restored the sensitivity to TAM and reduced the frequency and stemness in TAM‐resistant breast cancer cells. Taken together, our data indicate that down‐regulated DLG5 expression increases the stemness of breast cancer cells by enhancing TAZ expression, contributing to TAM resistance in breast cancer.

## 1 INTRODUCTION

Estrogen receptor (ER) positive breast cancers account for the majority (70%‐80%) of breast cancers.^1^, ^2^ Tamoxifen (TAM), an ER antagonist, is a first line of therapeutic drug for pre‐menopausal patients with ER^+^ breast cancer. Unfortunately, high frequency of TAM resistance remains a risk for metastasis and progression of ER+ breast cancers.^3^, ^4^ Breast cancer stem cells (BCSCs) are thought to be critical players for drug resistance.^5^, ^6^ BCSCs can regulate the sensitivity of breast cancer cells to TAM by modulating the NF‐κB, PI3K/PTEN/AKT/mTOR, β‐catenin and HER2 pathways.^7^, ^8^ Therefore, reducing the frequency of BCSCs and inhibiting their function will be valuable for control of TAM resistance.

Discs large homolog 5 (DLG5) is a member of the membrane‐associated guanylate kinase (MAGUK) family, and a necessary component for the formation and maintenance of epithelial tube and cell polarity.^9^ A recent study has showed that DLG5 can inhibit the progression of ER^+^ breast cancers and DLG5 deficiency promotes the migration and proliferation of ER^+^ breast cancer cells.^10^ DLG5 expression is upregulated in Luminal type of breast cancer tissues and positively correlated with ER expression. It is notable that DLG5 expression is down‐regulated in CD44^+^/CD24^−^ BCSCs.^11^ Our previous study and those of others have revealed that DLG5 silencing promotes the process of epithelial‐to‐mesenchymal transition (EMT) and stemness of ER^+^ breast cancer cells by inhibiting the Hippo signalling.^5^, ^10^, ^12^ However, how DLG5 regulates the stemness of breast cancer cells and TAM resistance has not been clarified. The Hippo signalling core kinases MST1/2 and downstream LATS1/2 phosphorylate YAP/TAZ, and promote their cytoplasmic sequestration and degradation.^13^, ^14^ Inhibition of MST1/2 and LATS1/2 by DLG5 silencing promotes the nuclear localization of YAP.^10^ Furthermore, TAZ, a YAP paralogue, is crucial for BCSCs.^15^, ^16^ Accordingly, we have been suggested that DLG5 may regulate TAM sensitivity by changing TAZ expression and nuclear translocation to modulate the stemness of breast cancer cells.

In this study, we examined DLG5 expression in paired of TAM‐sensitive and resistant breast cancer tissues and employed TAM‐sensitive and resistant ER^+^ breast cancer cells to determine the effect of altered DLG5 expression on TAM sensitivity, apoptosis and stemness as well as TAZ expression. Furthermore, we tested the impact of TAZ silencing on the TAM sensitivity and stemness of TAM‐resistant breast cancer cells. Our data indicated that down‐regulated DLG5 expression increased the breast cancer stem cell‐like characteristics by enhancing TAZ expression, contributing to TAM resistance in ER^+^ breast cancer.

## 2 MATERIALS AND METHODS

### 2.1 Patients and tissue specimens

The study was conducted, according to the Code of Ethics of the World Medical Association. The experimental protocol was approved by the Ethics Review Committee of the First Affiliated Hospital of Xi'an Jiaotong University. A total of 9 metastatic breast cancer tissues from patients, who were resistant to TAM, and their matched primary tumours were obtained from the First Affiliated Hospital of Xi'an Jiaotong University.^8^

### 2.2 Immunohistochemistry

The paraffin‐embedded breast cancer tissue sections (4 μm) were deparaffinized, rehydrated and subjected to antigen retrieval in sodium citrate buffer. The sections were treated with 3% hydrogen peroxide to inactivate endogenous peroxidase and treated with 10% goat serum in TBST at 37°C for 30 minutes. After being washed, the sections were incubated with antibodies against DLG5 (1:200, Abcam) or TAZ (1:200, Cell Signaling Technology) at 4°C overnight. Subsequently, the bound antibodies were reacted with biotinylated goat anti‐rabbit IgG (ZSGB‐Bio) and horseradish peroxidase (HRP)‐conjugated streptavidin. After being washed, the stained signals were visualized with diaminobenzidine and counterstained with hematoxylin. The signals were photographed using a microscope slide scanner (Leica MP, SCN400). The intensity of antibody staining in individual fields was semi‐quantitatively analysed, as described previously.^8^

### 2.3 Cell culture, infection and transfection

Human ER+ breast cancer MCF7 cells were given by Dr. Jianmin Zhang (Roswell Park Cancer Institute, Buffalo, New York, USA) and cultured in DMEM (Hyclone) supplemented with 10% fetal bovine serum (FBS) (Hyclone). The TAM‐resistant MCF7‐TamR and LCC2 cells were derived from MCF7 by continuous exposure to TAM, as previously described^8^ and cultured in phenol‐red‐free DMEM supplemented with 5% charcoal‐dextran stripped FBS and 1 μmol L^−1^ 4‐hydroxytamoxifen (4‐OHT) (Sigma). MCF7 and LCC2 cells were transduced with lentivirus for expression of shDLG5 or DLG5 to generate DLG5‐silenced MCF7 (MCF7‐shDLG5) and DLG5 overexpression LCC2 (LCC2‐oxDLG5), respectively.^10^ In addition, MCF7 cells were transfected with scramble control or TAZ‐specific siRNA using lipofectamine 2000. The sequences of TAZ‐specific siRNAs were TAZ‐1 sense, 5′‐GCU CAU GAG UAU GCC CAA UTT‐3′ and TAZ‐2 sense, 5′‐CCU CAA UGG AGG GCC AUA UTT‐3′.

### 2.4 Quantitative real‐time PCR (qRT‐PCR)

Total RNA was extracted from individual groups of cells using trizol and reversely transcribed into cDNA using the High Capacity cDNA Reverse Transcription Kit (Takara Biotechnology), according to the manufacturer's instruction. The relative levels of DLG5 to control GAPDH mRNA transcripts were determined qRT‐PCR using the SYBR^®^ Green Real‐Time PCR Master Mix kit (Thermofisher) and specific primers (Takara) in the QuantStudio 6 Flex Real‐time PCR system (Applied Biosystems). The sequences of primers were GAPDH forward 5′‐CTC CTC CAC CTT TGA CGC TG‐3′ and reverse 5′‐TCC TCT TGT GCT CTT GCT GG‐3′; DLG5 forward 5′‐CTG CAC ATC AAC CTC AGT GG‐3′ and reverse 5′‐CGG CAG CAT ACA CTC CATT‐3′. The results were analysed by 2^−ΔΔCt^.

### 2.5 Western blot

The different groups of cells were lysed by RIPA buffer supplemented with protease inhibitors and phosphatase inhibitors (Roche). After quantification with BCA reagents, individual cell lysates (30 μg/lane) were separated by sodium dodecyl sulfate polyacrylamide gel electrophoresis SDS‐PAGE on 10% gels and transferred onto polyvinylidene difluoride (PVDF) membranes (Millipore). The membranes were treated with 5% fat‐free dry milk in TBST and incubated with anti‐DLG5, anti‐TAZ, anti‐β‐catenin, anti‐Oct4, anti‐c‐MYC (Cell Signaling Technology), anti‐p‐TAZ (Santa Cruz Biotechnology) and anti‐GAPDH (Proteintech) overnight at 4°C. After being washed, the bound antibodies were detected with HRP‐conjugated second antibodies and visualized using the enhanced chemiluminescent (ECL) Plus reagents (Millipore). The relative levels of target protein to the control GAPDH were determined by densitometric analysis using the Image J software.

### 2.6 Cell viability assay

The different groups of cells were cultured in 96‐well plates and treated in triplicate with 4‐OHT (0, 2, 5 mol L^−1^) for 24, 48 and 72 hours. During the last 4 hours culture, individual wells of cells exposed to 5 mg/mL of MTT reagent. The generated formazan in individual wells was dissolved in 200 μL DMSO and the absorbance was measured at 570 nm in a microreader.

### 2.7 Immunofluorescence

The different groups of cells were fixed with a 4% paraformaldehyde for 15 minutes at room temperature (RT) and permeabilized in 0.2% Triton X‐100 in PBS for 10 minutes. After being blocked with 5% bovine serum albumin (BSA) and 10% horse sera in PBS for 1 hour at RT, the cells were incubated with antibodies against β‐catenin and TAZ (Cell Signal Technology) at 4°C overnight. The bound antibodies were detected with Alexa Fluor 488‐conjugated secondary antibodies (Invitrogen) and co‐stained with DAPI. Fluorescent signals were captured under a confocal laser scanning microscope (Leica SP5II).

### 2.8 Flow cytometry analysis

The different groups of cells were harvested and stained with APC‐anti‐CD44 and PE‐anti‐CD24 (Biolegend). The ALDH^+^ cells were characterized using the ALDEFLUOR kit (Stem Cell Technologies), according to the manufacturer's instruction. The cells were analysed by flow cytometry (BD FACS Canto II) and analysed by FlowJo software, as described previously.^17^ In addition, the different groups of cells were treated in triplicate with vehicle or 5.0 mol L^−1^ 4‐OHT for 48 hours and stained with PE‐Annexin V and 7‐AAD. The percentages of apoptotic cells were determined by flow cytometry.

### 2.9 Mammosphere formation assay

The different groups of cells (10^4^ cells/well) were cultured in FBS‐free DMEM:F12 media (Gibco) supplemented with 20 μL/mL of B27 (Invitrogen) and 20 ng/mL epithelial growth factor (EGF, Peprotech) in ultralow attachment 6‐well plates (Corning). The cells were exposed to half volume of fresh medium (500 μL) every 3 days. On day 10 after incubation, the formed mammospheres in individual wells were captured under a light microscope.

### 2.10 Soft‐agar assay

The clonogenicity of individual groups of cells was determined by soft‐agar colony formation assay, as described previously.^17^ Briefly, individual groups of cells (2.5 × 10^3^ cells/well) were mixed with 0.3% agarose in medium and cultured in 6‐well plates that had been coated with 0.8% agarose in medium for 20 days. The formed cell clones were stained by 0.02% iodonitrotetrazolium chloride (Sigma‐Aldrich). The numbers of colonies were counted in a blinded manner.

### 2.11 Statistical analysis

Data are expressed as the mean ± standard deviation (SD). The difference between groups was analysed by Student's *t* test using GraphPad Prism Version 7.00. A *P‐value* of <0.05 was considered statistically significant.

## 3 RESULTS

### 3.1 Down‐regulated DLG5 expression in TAM‐resistant breast cancer tissues and cells

**Figure 1:**
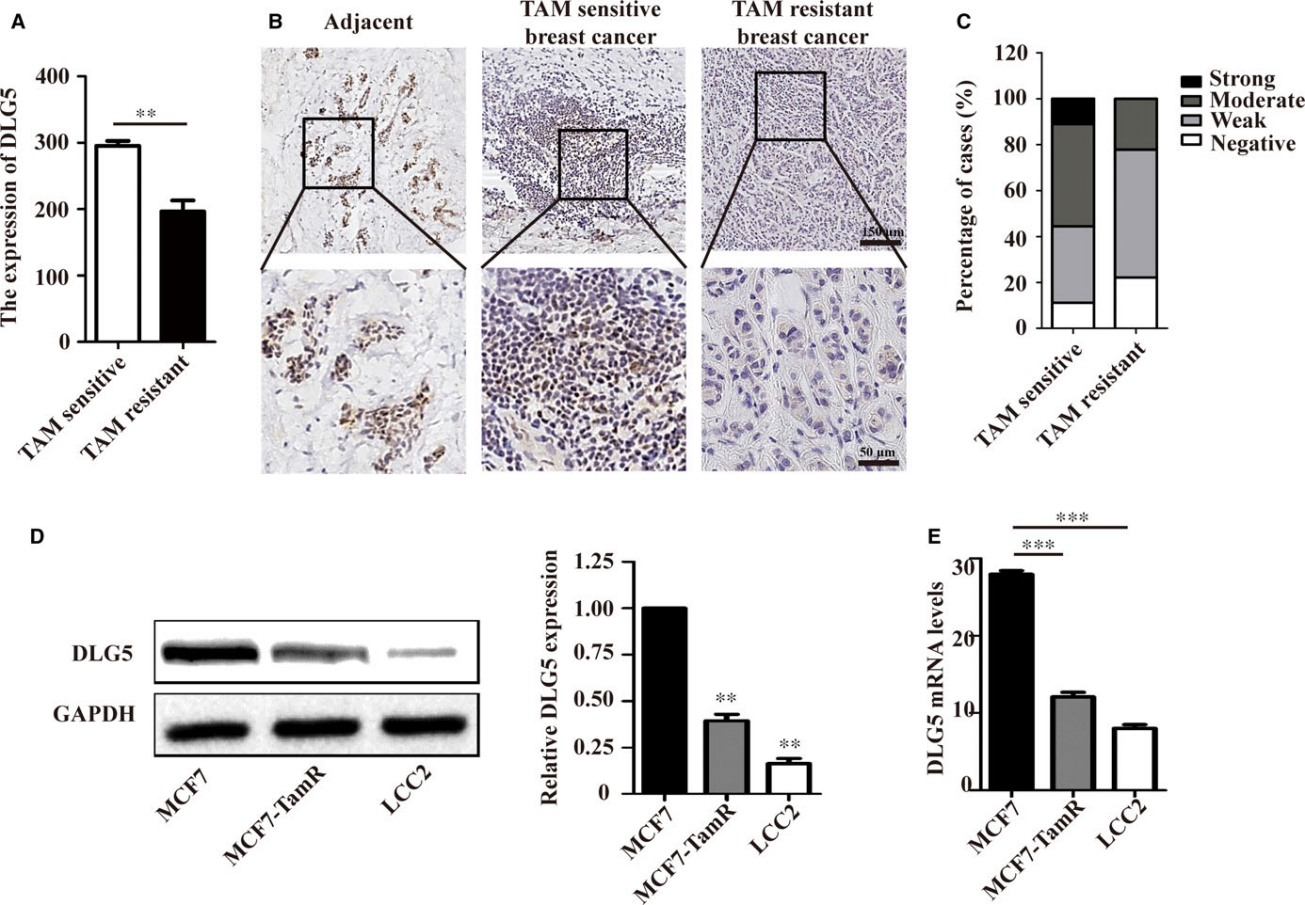
Down‐regulated DLG5 expression is associated with the TAM resistance in breast cancer. (A) The levels of *DLG5* expression were analysed by a gene expression profile in the GEO database (GSE26459); (B, C) Immunohistochemistry analysis of DLG5 protein expression in nine paired of adjacent non‐tumour breast, TAM‐sensitive and resistant breast cancer tissues. (D, E) Western blot and qRT‐PCR analysis of DLG5 expression in MCF7, MCF‐TamR and LCC2 cells; Data are representative images (magnification ×200 upper panels, 400 bottom panels) or expressed as the mean ± SD of each group from three separate experiments. **P < 0.01, ***P < 0.001

The relationship between DLG5 expression and TAM resistance in ER^+^ breast cancer was firstly analysed by a gene expression profile in the GEO database (GSE26459), which showed that *DLG5* expression in the TAM‐resistance MCF7 cells was significantly lower than that in the TAM‐sensitive MCF7 cells (*P* < 0.01, Figure 1A). Immunohistochemistry analysis of nine paired of TAM resistant and sensitive breast cancer tissues indicated that while higher levels of DLG5 expression were detected in adjacent tissues the levels of DLG5 expression in the TAM‐resistant breast cancer tissues were lower than that in the TAM‐sensitive breast cancer tissues in this population (Figure 1B and C, Table 1). Similarly, the relative levels of DLG5 mRNA transcripts and protein expression in the TAM‐resistant MCF7‐TamR cells were significantly lower than that in the TAM‐sensitive MCF‐7 cells, but higher than that in the high TAM‐resistant LCC2 cells (Figure 1D‐E). Such data indicated that down‐regulated DLG5 expression was associated with TAM resistance in breast cancer.

**Table 1:** Immunohistochemistry analysis of DLG5 protein expression in nine paired of TAM‐sensitive and resistant breast cancer tissues

	Negative <25%	Weak 25%‐50%	Moderate 50%‐75%	Strong >75%
TAM sensitive	1 (11.1)	3 (33.3)	4 (44.4)	1 (11.1)
TAM resistant	2 (22.2)	5 (55.6)	2 (22.2)	0

### 3.2 Altered DLG5 expression modulates the sensitivity of breast cancer cells to TAM

**Figure 2:**
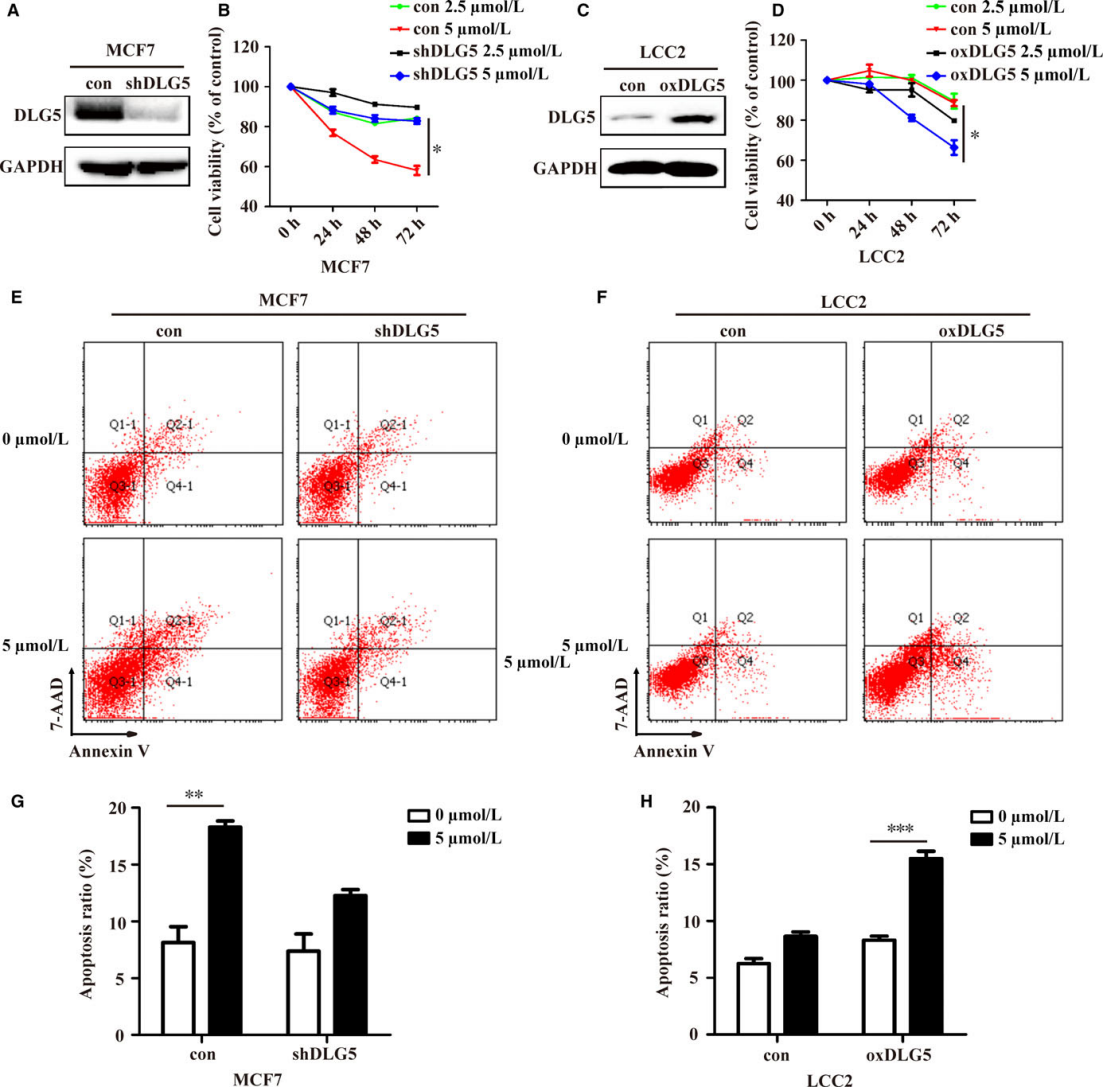
Altered DLG5 expression changes the sensitivity of breast cancer cells to TAM in vitro. MCF7 and LCC2 cells were transduced with lentivirus for DLG‐specific shRNA and DLG5 overexpression, respectively and the levels of DLG5 expression were determined by Western blot (A, C). The sensitivity of different groups of cells to 2.5 or 5.0 μmol L^−1^ 4‐OHT was determined longitudinally by MTT (B, D). Subsequently, the cells were treated with vehicle or 5.0 μmol L^−1^ 4‐OHT for 48 hours and the percentages of apoptotic cells were determined by flow cytometry following PE‐Annexin V and 7‐AAD staining (E‐H). Data are representative images or expressed as the mean ± SD of each group from three separate experiments. *P < 0.05, **P < 0.01, ***P < 0.001

To understand the role of DLG5 in regulating the sensitivity of breast cancer cells to TAM, the high levels of DLG5 expressing MCF7 and low levels of DLG5 expressing LCC2 cells were transduced with lentivirus for DLG5 silencing or overexpression, respectively. Transduction with DLG5‐specific shRNA significantly attenuated DLG5 expression in MCF7 cells (Figure 2A) and reduced their sensitivity to 4‐OHT (Figure 2B). In contrast, transduction with lentivirus for DLG5 expression dramatically increased the levels of DLG5 expression in LCC2 cells (Figure 2C) and their sensitivity to 4‐OHT (Figure 2D). Furthermore, DLG5 silencing significantly mitigated the 4‐OHT (5 mol L^−1^)‐mediated apoptosis in MCF7 cells while DLG5 overexpression enhanced the same dose of 4‐OHT‐triggered apoptosis of LCC2 cells (Figure 2E‐H). Hence, altered DLG5 expression modulated the sensitivity of breast cancer cells to TAM in vitro.

### 3.3 DLG5 reduces the frequency of breast cancer stem‐like cells

**Figure 3:**
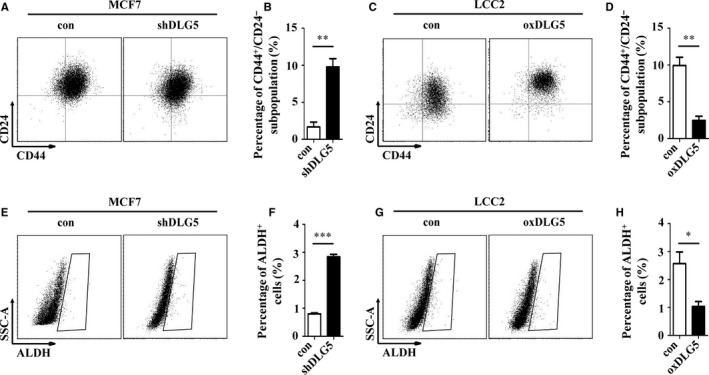
DLG5 reduces the frequency of CD44^+^/CD24^−^ BCSCs in breast cancer cells. The percentages of CD44^+^/CD24^−^ BCSCs or ALDH ^+^ cells in MCF7, MCF7‐shDLG5, LCC2 and LCC2‐oxDLG5 cells were determined by flow cytometry. Data are representative images or expressed as the mean ± SD of each group from three separate experiments. (A, B) The frequency of CD44^+^/CD24^−^ BCSCs in MCF7 cells. (C, D) The frequency of CD44^+^/CD24^−^ BCSCs in LCC2 cells. (E, F) The frequency of ALDH ^+^ cells in MCF7 cells. (G, H) The frequency of ALDH ^+^ cells in LCC2 cells. *P < 0.05, **P < 0.01, ***P < 0.001

**Figure 4:**
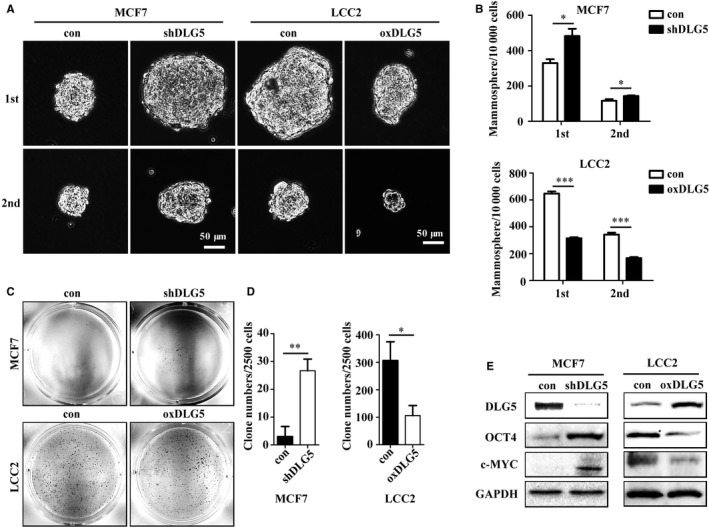
DLG5 reduces the breast cancer cell stemness in vitro. The formation of mammospheres and clones of MCF7, MCF7‐shDLG5, LCC2 and LCC2‐DLG5 cells were determined by mammosphere formation and soft‐agarose colony formation assays, respectively. The relative levels of DLG5, Oct4 and c‐MYC to GAPDH were determined by Western blot. Data are representative images or expressed as the mean ± SD of each group from three separate experiments. (A, B) The mammosphere formation. Scale bar, 50 μm. (C, D) The colony formation. (E) Western blotting analysis of DLG5, OCT4 and c‐MYC expression in the different groups of cells

CD44^+^/CD24^−^ BCSCs have stemness property and are associated with drug resistance.^18^ Next, we investigated whether DLG5 could modulate the frequency of BCSCs, contributing to its enhanced sensitivity to TAM. Flow cytometry analysis indicated that DLG5 silencing significantly increased the percentages of CD44^+^/CD24^−^ BCSCs in MCF7 cells (*P* < 0.01, Figure 3A and B) while DLG5 overexpression decreased the frequency of CD44^+^/CD24^−^ BCSCs in LCC2 cells. A similar pattern of ALDH^+^ MCF7 and ALDH^+^ LCC2 cells was detected in the different groups of cells (Figure 3E‐H). Given that BCSCs can form mammospheres in a non‐adherent non‐differentiating condition^19^ we further characterized the effect of altered DLG5 expression on the mammosphere formation of breast cancer cells in vitro. DLG5‐silencing significantly increased the numbers and size of primary and secondary mammospheres of MCF7 cells while DLG5 overexpression decreased the numbers and size of primary and secondary mammospheres of LCC2 cells (*P* < 0.05 or *P* < 0.001, Figure 4A and B). Further soft‐agar colony formation assays revealed that DLG5 silencing increased the numbers of cell clones in MCF7 cells while DLG5 overexpression significantly decreased the numbers of form clones in LCC2 cells (*P* < 0.05 or *P* < 0.01, Figure 4C and D). Western blot displayed that DLG5 silencing dramatically enhanced the relative levels of Oct4 and c‐MYC expression in MCF7 cells while DLG5 overexpression reduced the relative levels of Oct4 and c‐MYC expression in LCC2 cells (Figure 4E). Collectively, such data demonstrated that DLG5 reduced the frequency of CD44^+^/CD24^−^ BCSCs.

### 3.4 DLG5 suppresses the breast cancer stem cell‐like characteristics to restore TAM sensitivity by inhibiting TAZ expression and nuclear translocation

**Figure 5:**
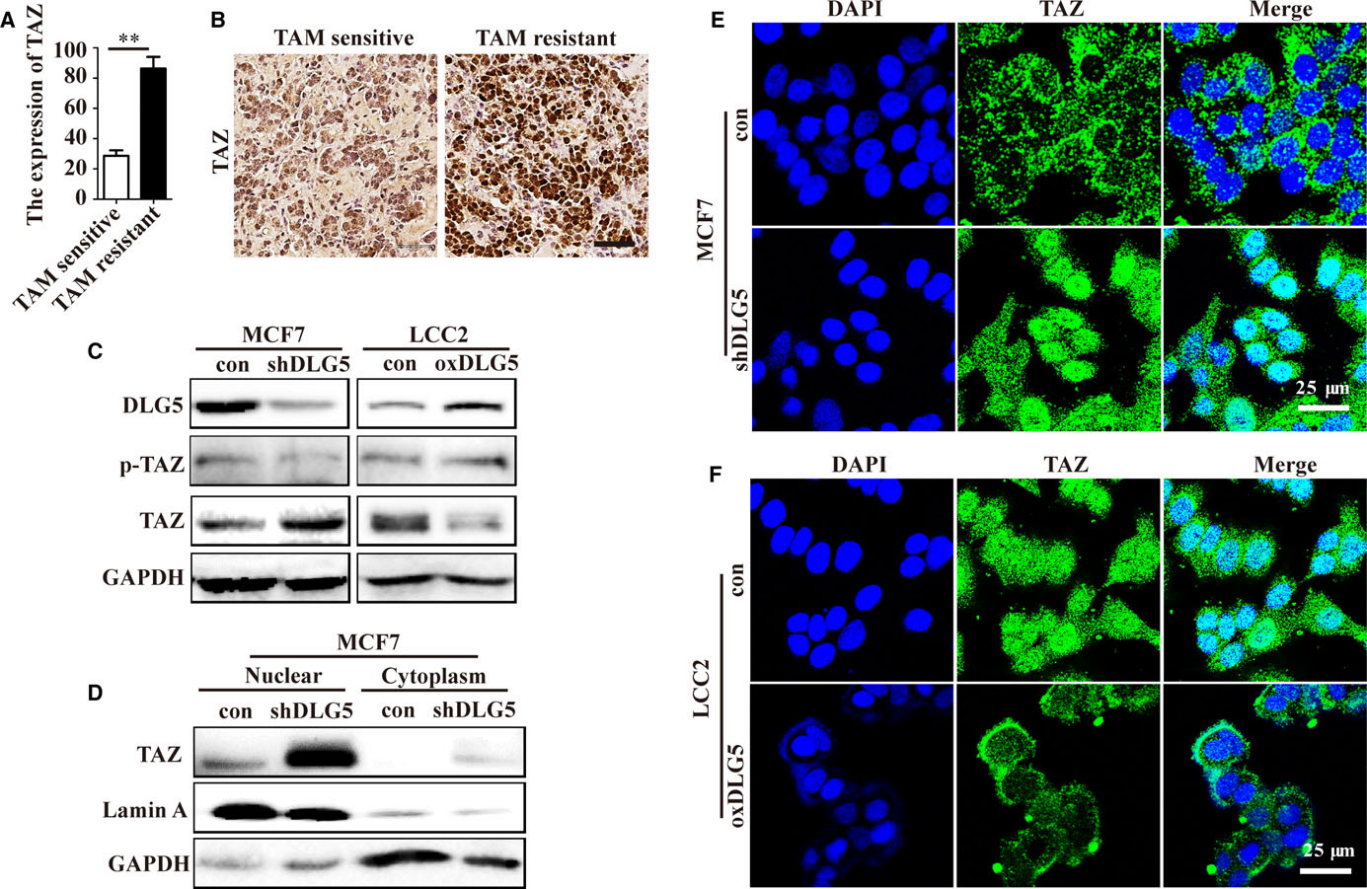
Down‐regulated DLG5 expression promotes TAZ expression and nuclear localization in breast cancer cells. (A) Analysis of *TAZ* expression in the GEO database (GSE26459). (B) Immunohistochemistry analysis of TAZ expression in TAM‐sensitive and resistant breast cancer tissues. Scale bar, 50 μm (C) Western blot analysis of TAZ expression and phosphorylation in the indicated cells. (D) Western blot analysis of TAZ expression in the cytoplasm and nuclei of breast cancer cells. (E, F) Immunofluorescent analysis of TAZ protein distribution in breast cancer cells. Scale bar, 25 μm. Data are representative images or expressed as the mean ± SD of each group from three separate experiments. **P < 0.01

Given that the Hippo transducer TAZ is required for sustained self‐renewal of BCSCs,^16^ we analysed *TAZ* expression in the gene expression profile from the GEO database (GSE26459) and our paired breast cancer tissues. The results indicated that the relative levels of *TAZ* expression in TAM‐resistant MCF7 cells were significantly higher than that in the TAM‐sensitive MCF7 (Figure 5A and B). Similarly, DLG5 silencing decreased the TAZ phosphorylation, and increased the relative levels of TAZ and its nuclear translocation in MCF7 cells while DLG5 overexpression increased the TAZ phosphorylation, and decreased TAZ protein expression, and nuclear translocation in LCC2 cells (Figure 5C and D). A similar pattern of TAZ expression and nuclear translocation was detected in the different groups of cells by immunofluorescent assays (Figure 5E and F). Hence, DLG5 inhibited TAZ expression and nuclear translocation in breast cancer cells.

**Figure 6:**
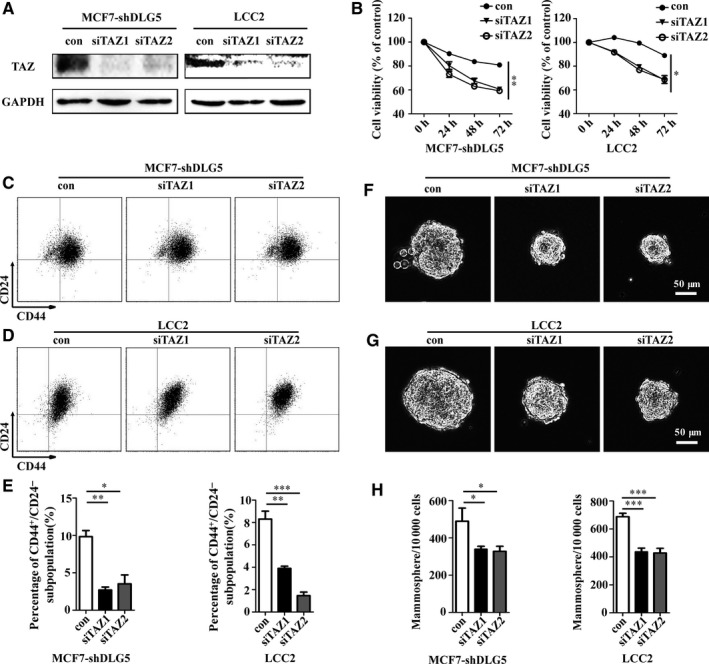
TAZ silencing restores TAM sensitivity in TAM‐resistant breast cancer cells by inhibiting the breast cancer stemness. TAM‐resistant MCF7‐shDLG5 and LCC2 cells were transfected with control scramble or TAZ‐specific siRNA. The relative levels of TAZ expression were determined by Western blot (A). The sensitivity of different groups of cells to 5 mol L^−1^ 4‐OHT was analysed by MTT (B). The frequency of CD44^+^/CD24^−^ BCSCs was determined by flow cytometry (C‐E). The formation of mammospheres was examined by mammosphere formation assays (F‐H). Scale bar, 50 μm. Data are representative images or expressed as the mean ± SD of each group from three separate experiments. *P < 0.05, **P < 0.01, ***P < 0.001

To verify the relationship among DLG5, TAZ and TAM resistance, the TAM resistant MCF7‐shDLG5 and LCC2 cells were transfected with control scramble or TAZ‐specific siRNA to silence TAZ expression (Figure 6A). Furthermore, TAZ‐silencing significantly enhanced the sensitivity of TAM‐resistant MCF7‐shDLG5 and LCC2 cells to TAM (Figure 6B). TAZ‐silencing significantly decreased the frequency of CD44^+^/CD24^−^ BCSCs in MCF7‐shDLG5 and LCC2 (Figure 6C‐E). TAZ‐silencing prominently decreased the numbers and size of formed mammospheres in MCF7‐shDLG5 and LCC2 (Figure 6F‐H). Together, such data indicated that down‐regulated DLG5 promoted TAM resistance of breast cancer by promoting TAZ expression and nuclear translocation.

## 4 DISCUSSION

TAM has been widely used for treatment of ER^+^ breast cancers in the clinic.^20^ However, TAM resistance is a huge challenge for clinical practice, and promotes breast cancer metastasis and death.^21^, ^22^ Our previous studies and those of others have shown that DLG5 acts as a tumour suppressor in breast cancer, and its expression is up‐regulated in Luminal type, but not basal‐like, breast cancer.^10^, ^11^ *DLG5* is a primary target of progesterone receptor^23^ and DLG5 expression is positively correlated with ER and PR expression in breast cancers.^10^ Loss of DLG5 expression induces EMT and disrupts epithelial cell polarity, which are associated with altered expression of cell polarity proteins, such as Scribble, ZO1, E‐cadherin and N‐cadherin and their mislocalization.^10^ Furthermore, DLG5 expression is down‐regulated in CD44^+^/CD24^−^ breast cancer stem cell‐like characteristics cells.^11^ Together, the decreased DLG5 expression usually occurs in the breast cancer cells and tissues with the characteristics, such as lower ER expression, loss of cell polarity, enhanced EMT process or increased CD44^+^/CD24^−^ phenotype. Accordingly, loss of ER expression or cell‐cell junctions, undergoing EMT process and increased BCSC cells occur in TAM resistant tumours.^5^, ^24^, ^25^ In this study, we found a down‐regulated DLG5 expression in TAM‐resistant breast cancer tissues and cells. Induction of DLG5 overexpression restored the TAM sensitivity of LCC2 cells. Our findings support the notion that DLG5 enhances the sensitivity to TAM in ER^+^ breast cancer.

Currently the precise mechanisms underlying the TAM resistance are not fully understood. Previous studies have indicated that the potential mechanisms underlying TAM resistance mainly include (a) loss of ERα expression and function; (b) alteration in the levels of ERβ expression; (c) pharmacogenomic effects and pharmacological interactions may alter the metabolism and efficacy of TAM; (d) alterations in the expression of co‐regulatory proteins of ER, such as AIB1, HDACs and NF‐κB; (e) alternations in the cellular kinase and signal pathways, such as the IGFR, EGFR/ERBB2, MAPK and BCAR1; (f) endocrine adaptation (just in a minority of patients)^26^, ^27^ In additional, increased stemness and cancer stem cell‐like characteristics can contribute to drug resistance.^5^, ^6^, ^7^ CSCs can self‐renew and differentiate to different types of mature cancer cells, leading to the development and progression of malignant tumours.^28^, ^29^ BCSCs are major players of drug resistance and can promote the development of TAM resistance in breast cancer by increasing their stemness.^7^, ^30^, ^31^ In this study, we found that DLG5 silencing increased the frequency of CD44^+^/CD24^−^ and ALDH^+^ BCSCs, the formation of mammospheres, anchorage‐independent growing clonogenicity and Oct4 and c‐MYC expression in ER^+^ MCF7 cells while induction of DLG5 overexpression decreased them in TAM‐resistant LCC2 cells. Such data were similar to a previous report that DLG5 expression was reduced in CD44^+^/CD24^−^ population in MCF10A cells.^11^ Given that down‐regulated DLG5 expression was associated with TAM resistance in breast cancer our findings suggest that DLG5 may inhibit TAM resistance by attenuating the stemness of BCSCs.

Previous studies have suggested that BCSCs promote TAM resistance by modulating the Wnt/β‐catenin, Notch, PI3K/PTEN/AKT/mTOR and NF‐κB signalling as well as changing the HER2 and SOX2 expression in breast cancer.^7^, ^8^ Furthermore, TAZ, a transducer of the Hippo signalling, can support the self‐renewal and stemness of BCSCs.^16^ A previous study has shown that DLG5 expression is negatively associated with the expression of YAP, a paralogue of TAZ in the Hippo signal pathway.^11^ Actually, DLG5 silencing attenuates the Hippo signal pathway by inhibition the interaction of Mst1/2 and Lats1 with Scribble, consequently increasing the expression and nuclear localization of YAP.^10^ In this study, we first found that TAZ expression was up‐regulated in TAM‐resistant breast cancer cells and DLG5 silencing enhanced TAZ expression and nuclear translocation in TAM‐resistant breast cancer cells. Moreover, TAZ silencing restored the sensitivity to TAM and decreased the stemness of TAM‐resistant breast cancer cells. Given that up‐regulated YAP expression is associated with TAM resistance, dependent on the HSP90‐HDAC6 regulating network.^32^ Given that YAP and TAZ are the paralogue and play a similar role in organ size control, tissue homeostasis and cancer,^14^, ^33^, ^34^ our findings suggest that TAZ, like YAP, may promote TAM resistance. Hence, our findings may provide new insights into the molecular mechanisms underlying the TAM resistance. Conceivably, the inhibition of TAZ or the enhancement of DLG5 expression may be valuable for the restoration of TAM sensitivity in TAM‐resistant ER^+^ breast cancers.

In summary, our data indicated that DLG5 expression was down‐regulated in ER^+^ TAM‐resistant breast cancer tissues and cells, and DLG5 silencing increased the resistance to TAM and breast cancer cell stemness by enhancing TAZ expression and nuclear translocation in ER^+^ breast cancer cells. Furthermore, TAZ silencing restored the sensitivity to TAM and inhibited the stemness in TAM‐resistant ER^+^ breast cancer cells. Therefore, DLG5 and TAZ may be valuable therapeutic targets for control of TAM resistance in ER^+^ breast cancer. Our findings may provide new insights into the regulation of DLG5 on BCSC‐related TAM resistance. We are interested in further investigating the molecular mechanisms underlying the action of DLG5 in regulating TAZ expression in ER^+^ breast cancer.

## CONFLICT OF INTEREST

The authors declare no conflicts of interest.

## ETHICS APPROVAL

All procedures performed in this study involving human participants were in accordance with the ethical standards of the institutional and/or national research committee and with the 1964 Helsinki declaration and its later amendments or comparable ethical standards.

## INFORMED CONSENT

Written informed consent was obtained from all individual participants included in this study.
